# An Advanced Method to Assess the Diet of Free-Ranging Large Carnivores Based on Scats

**DOI:** 10.1371/journal.pone.0038066

**Published:** 2012-06-08

**Authors:** Bettina Wachter, Anne-Sophie Blanc, Jörg Melzheimer, Oliver P. Höner, Mark Jago, Heribert Hofer

**Affiliations:** 1 Evolutionary Ecology, Leibniz Institute for Zoo and Wildlife Research, Berlin, Germany; 2 Institute of Zoology, University of Neuchâtel, Neuchâtel, Switzerland; 3 The AfriCat Foundation, Otjiwarongo, Namibia; University of Western Ontario, Canada

## Abstract

**Background:**

The diet of free-ranging carnivores is an important part of their ecology. It is often determined from prey remains in scats. In many cases, scat analyses are the most efficient method but they require correction for potential biases. When the diet is expressed as proportions of consumed mass of each prey species, the consumed prey mass to excrete one scat needs to be determined and corrected for prey body mass because the proportion of digestible to indigestible matter increases with prey body mass. Prey body mass can be corrected for by conducting feeding experiments using prey of various body masses and fitting a regression between consumed prey mass to excrete one scat and prey body mass (correction factor 1). When the diet is expressed as proportions of consumed individuals of each prey species and includes prey animals not completely consumed, the actual mass of each prey consumed by the carnivore needs to be controlled for (correction factor 2). No previous study controlled for this second bias.

**Methodology/Principal Findings:**

Here we use an extended series of feeding experiments on a large carnivore, the cheetah (*Acinonyx jubatus*), to establish both correction factors. In contrast to previous studies which fitted a linear regression for correction factor 1, we fitted a biologically more meaningful exponential regression model where the consumed prey mass to excrete one scat reaches an asymptote at large prey sizes. Using our protocol, we also derive correction factor 1 and 2 for other carnivore species and apply them to published studies. We show that the new method increases the number and proportion of consumed individuals in the diet for large prey animals compared to the conventional method.

**Conclusion/Significance:**

Our results have important implications for the interpretation of scat-based studies in feeding ecology and the resolution of human-wildlife conflicts for the conservation of large carnivores.

## Introduction

Diet is an important part of carnivore ecology and conservation. Information on diet composition is needed when predator-prey relationships are studied [1], when predators are perceived as a threat to livestock of farmers [2] or when rare prey species may need to be protected [3]. Due to the elusive behaviour of many carnivore species and the small chances of finding fresh kills, indirect methods of determination of carnivore diets based on indigestible prey remains in scats such as hairs, bones, teeth, hooves and claws is often the most appropriate, accurate and feasible method. This method allows the determination of the range of prey species consumed by carnivores, the frequency at which remains of prey species occur in scats and the proportion each prey species contributes to the diet. The latter, which is often the information needed, cannot be calculated directly from the frequency at which remains of prey species occur in scats because smaller animals have a higher surface-to-volume (hair-to-meat) ratio than larger animals [4]. This affects the production of scats: When carnivores feed from a small prey animal, they consume less prey mass to excrete one scat than when they feed from a large prey animal [2], [5], [6]. This is consistent with the finding that smaller animals are less digestible than larger animals [7]. As a result, the frequency of occurrence of prey species in scats is likely to over-represent smaller prey animals in terms of consumed prey mass in the diet [4].

**Figure 1:**
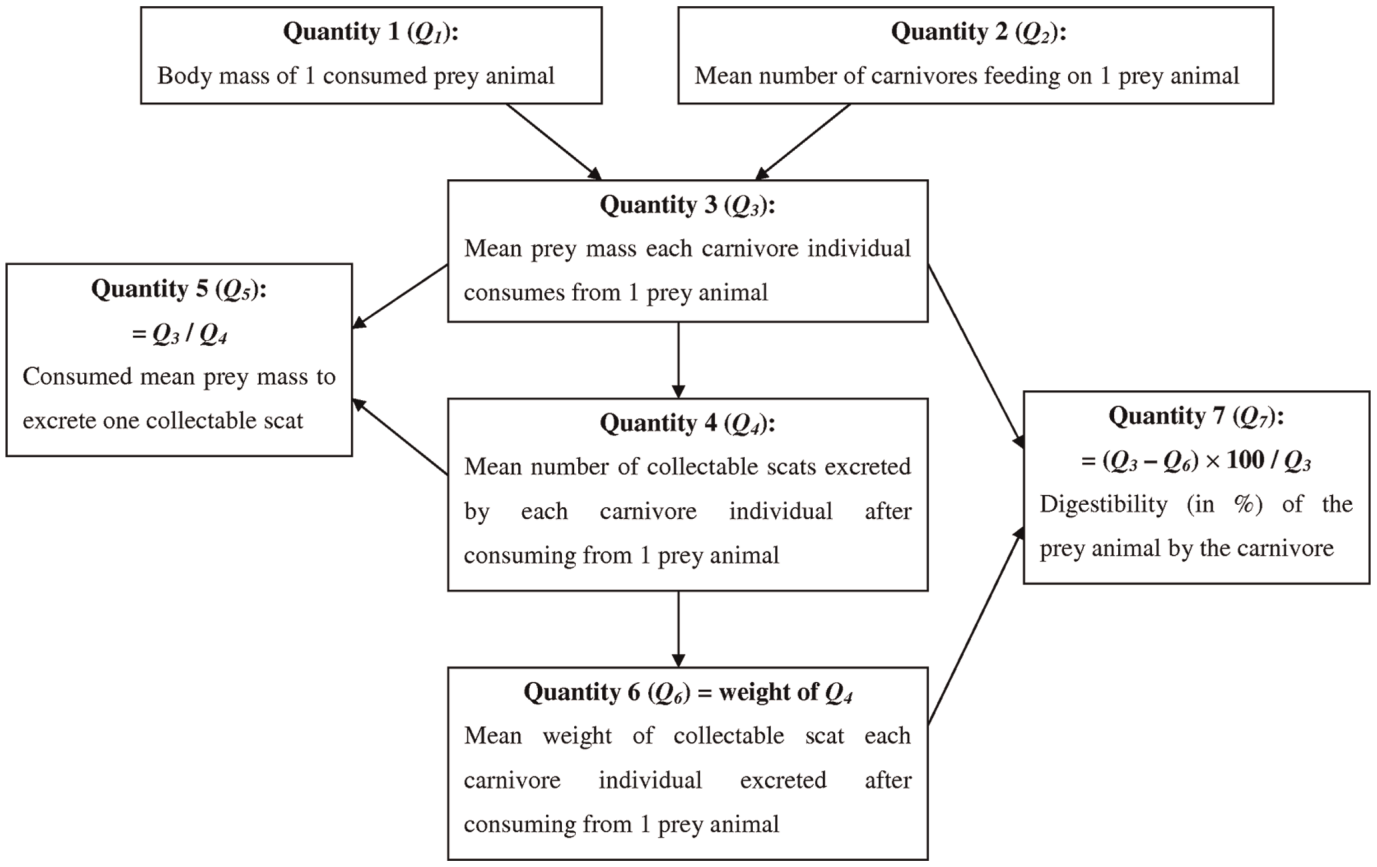
The seven quantities derived from carnivore scats. These quantities describe the conversion of prey body mass into numbers and weights of collectable scats excreted by a carnivore individual after prey consumption and the determination of prey digestibility.

The conversion of prey body mass into a number of scats and their weights excreted by an individual carnivore after prey consumption is a crucial step that diet studies based on scats ought to infer accurately. We use seven quantities to evaluate the steps required to establish the consumed prey mass and the number of consumed prey individuals derived from carnivore scats collected in the field (Fig. 1). By applying these quantities to different sampling schemes (scat collection schedules) and carnivore group sizes we demonstrate that previous procedures were not aware of an important source of bias and introduce a new correction factor to take this bias into account.

### Diet Expressed as Proportions of Consumed Prey Mass

To control for the influence of prey body mass (*Q_1_*) on the consumed prey mass to excrete one scat (*Q_5_*), previous studies established a correction factor ( = correction factor 1, CF1) using feeding experiments (wolves (*Canis lupus*): [5], [7]–[9], cougar (*Felis concolor*): [6], cheetah (*Acinonyx jubatus*): [2]). These studies applied the widely used ‘biomass model’ [5] which is based on a linear regression (*y* = a*x* + b) with *x* being the provided prey body mass (*Q_1_*) and *y* being the consumed prey mass to excrete one scat (*Q_5_*). The diet in terms of the proportions of consumed mass per species is then determined by multiplying the frequency of occurrence of prey species *i* identified in *n* collected scats (*n_i_*) by *y* for prey species *i* ( = *Q_5i_*) using the (reasonable) assumption of prey body mass *x* = *Q_1i_* [2], [5], [10]–[13]. Thus, the consumed mass of prey species *i* by a carnivore can be described as *n_i_*×*Q_5i_*. Other studies determined CF1 for a given prey species by calculating the ratio of consumed mass to dry mass of prey remains in scats and determined the diet in terms of the proportion of consumed mass per species using this CF1 (wolves: [14], [15], lynxes (*Lynx lynx*): [15], [16]).

Possibly because of providing a limited range of prey body masses, all previous studies that conducted feeding experiments to determine CF1 fitted a linear regression to their data [2], [5]–[9], [16]. A linear regression suggests that consumed prey mass to excrete one scat does not reach an asymptote for large prey body mass. We would expect that consumed prey mass to excrete one scat reaches such an asymptote because there will be a limit to the prey mass consumed by a carnivore which can be digested per excreted scat and there will be a limit to the size of scats that can be excreted. The regressions therefore should approach an asymptote, hence follow a non-linear function, which we assess to be physiologically more meaningful and realistic.

We evaluated this possibility in the cheetah, a species for which a previous study used a linear relationship between *y* and *x* [2]. We conducted a series of feeding experiments that included the entire range of body sizes of potential prey [17] as well as prey animals of sizes larger than a single cheetah is able to kill. Inclusion of prey of sizes that a carnivore may not be able to overcome alone but only in a group, or not even then, provides information on the overall physiological characteristics of the digestive system. Even if the predator in the wild only feeds from a fraction of the presented range of prey sizes of the feeding trials, the knowledge of the digestive properties for prey weights ranging across several orders of magnitude improves the accuracy of CF1 for any individual prey size. This is because the prey mass consumed per excreted scat for a particular prey size is a function of the overall physiological characteristics. Also for predator species mainly feeding on prey species smaller than its own body mass, it is advisable to use prey masses across a large range for the feeding experiment to allow a more accurate estimation of CF1 also for the small prey species. For carnivore species mainly feeding on fruits, invertebrates, birds and small mammals such as black-backed jackals (*Canis mesomelas*), side-striped jackals (*Canis adustus*) and red fox (*Vulpes vulpes*), we suggest that the specific conversion factors from feeding experiments with these food items should be used [18], [19], [20], [21] and diet determined following the extensive outline and review of [21].

### Diet Expressexd as Proportions of Consumed Individuals

Studies may not only be interested in the biomass of prey consumed by a carnivore but also, or primarily, in the number of prey individuals consumed [2], [5], [13], [15]. The number of consumed individuals of a given prey species *i* is commonly established by dividing the consumed mass of prey species *i*, which is *n_i_*×*Q_5i_*, by the entire body mass of the consumed prey species *i*, i.e. (*n_i_*×*Q_5i_*)/*Q_1i_*. Such calculations, however, are only appropriate when the carnivore consumes the prey animal entirely, but not when it consumes only part of it, as this leads to an underestimate of the numbers of large prey animals consumed. To our knowledge this issue has not been considered in previous studies [2], [5], [13], [15], although most large carnivores do not completely consume large prey [1], [2], [5], [22]. To account for this bias, the number of prey individuals in the diet should be calculated on the basis of the actual prey mass consumed (*Q_3_*) as suggested by [17]. The number of consumed individuals of prey species *i* is then equivalent to (*n_i_*×*Q_5i_*)/*Q_3i_* for solitary carnivores and ((*n_i_*×*Q_5i_*)/*Q_3i_*)/*Q_2_* for carnivores feeding in groups with a mean size of *Q_2_*. Because *Q_5i_* = *Q_3i/_Q_4i_*, *Q_5i_* can be substituted by *Q_3i/_Q_4i_*. The number of consumed individuals per prey species *i* is then simply *n_i/_Q_4i_* for solitary carnivores and (*n_i/_Q_4i_*)/*Q_2_* for carnivores feeding in groups with a mean size of *Q_2_*. *Q_4i_* is the number of scats excreted after consuming prey species *i* and can be derived from the feeding experiments using a regression between the number of excreted scats (*Q_4_*) and body mass (*Q_1_*). We termed this regression correction factor 2 (CF2).

These calculations are suggested if the prey species is large, the consumption of one individual (one feeding event) is likely to produce more than one scat and scats are collected in a defined area at regular intervals (daily, weekly or monthly) so that all detected scats excreted in the last couple of days or weeks are collected. Under the assumption that the likelihood of finding scats with remains of one feeding event of a given prey species is similar to the likelihood of finding scats with remains of different feeding events of the same prey species, scats containing remains of this prey species from one sampling interval can be pooled. Then, these scats should be treated as dependent samples and several scats assumed to represent one feeding event.

In contrast, if single carnivores of known identity are followed and observed to defecate or are immobilised and directly sampled, and if the sampling interval is longer than the time period during which a carnivore excretes scats after having fed on one prey animal, the scats are independent samples and cannot originate from one feeding event. In this case each scat containing remains of a particular prey species has to be multiplied by the mean number of scats a carnivore excreted after consuming from such a prey animal (*Q_4_*) to account for the missed scats of the carnivore individual. The number of consumed individuals per prey species is then equivalent to ((*n_i_*×*Q_4i_*)×*Q_5i_*)/*Q_3i_* for solitary carnivores and for carnivores feeding in groups since only one group member was sampled. Because *Q_5i_* = *Q_3i/_Q_4i_*, *Q_5i_* can be substituted by *Q_3i/_Q_4i_* and the number of consumed individuals per prey species for scats collected independently from each other is simply *n_i_*. It is important to be aware of whether scats collected in the field arise from one feeding event (dependent objects) or from independent feeding events, because the results and therefore the interpretation differ substantially.

In this study we introduce for the first time a CF1 based on a non-linear function and the new CF2 using a calibration study with cheetahs. We illustrate the application of both correction factors and contrast it with results obtained from the conventional method with a hypothetical example, and then proceed with calculations on the basis of both scat sampling schemes and for three different feeding group sizes. Using our protocol, we also derive CF1 and CF2 for other carnivore species from published data and apply them to published studies. We discuss the diet composition derived with the new method in comparison with results obtained from the conventional method.

## Methods

### Study Site

We conducted feeding experiments in north-central Namibia at AfriCat Foundation (20°51′S, 16°40′E). AfriCat Foundation is registered by the Namibian Ministry of Environment and Tourism (MET) as a large non-profit carnivore captive facility since 1993 (permit office 2004/11) and runs a rehabilitation centre for free-ranging carnivores, mainly cheetahs. AfriCat is equipped with high standard veterinary facilities and qualified staff caring for the animals.

### Ethics

Cheetahs are capable of killing a wide range of prey species. Reports range from hares (*Lepus sp*.) that weigh about 2 kg to adult zebra (*Equus burchelli*) that weigh up to 270 kg [23]. Cheetahs hunt either solitarily or in groups consisting of mother and cubs or male coalitions, with larger groups hunting larger prey animals [22]. Scat analyses of cheetahs further revealed that cheetahs also feed on prey species that are smaller than hares such as mice [17].

For the feeding experiments we used kudu (*Tragelaphus strepsiceros*), gemsbok (*Oryx gazella*), hartebeest (*Alcelaphus buselaphus*), springbok (*Antidorcas marsupialis*), warthog (*Phacochoerus africanus*), goat (*Capris sp*.), springhare (*Pedetes capensis*), ground squirrel (*Xerus inauris*), Namaqua rock mouse (*Aethomys namaquensis*) and hairy-footed gerbil (*Gerbillurus paeba*) as prey species. All large carcasses were animals killed by trophy hunters who booked their hunting with a registered professional Namibian hunting guide who had a hunting permit from the Ministry of Environment and Tourism (MET) in Windhoek, Namibia. Once the animal was shot in the field according to the hunting regulations from the NAPHA (Namibian Professional Hunting Association) and the trophy, i.e. head and horns, was removed, we retrieved the carcass. The smaller animals were trapped at the farms where our research stations are based. Commercial mouse traps of different sizes were used and once an animal was trapped it was quickly killed. Our study was approved by the MET (permit number 1089/2006), the scientific advisory board of the AfriCat Foundation and the leadership and Ethical Committee of the Leibniz Institute for Zoo and Wildlife Research in Berlin.

### Feeding Experiments

Between August 2006 and December 2006 we conducted fifteen feeding experiments on twelve cheetahs in four groups (two groups of four, one group of three and one single). During the experiments, the cheetahs were kept in observation enclosures of 20 m×10 m provided with water *ad libitum*. We provided cheetahs with prey animals weighing between 0.04 kg and 214 kg; we measured the mass of prey animals provided to and consumed by cheetahs in kg to an accuracy of 0.2 g using an electronic scale for prey animals up to 3.5 kg, and to an accuracy of 0.1 kg using a spring scale for larger prey animals. Large prey animals were fed to large groups to simulate natural feeding situations. For prey species smaller than springhare, more than one carcass was provided to avoid depriving the cheetahs of food (Table 1). In these cases, the cheetahs were given a carcass only after they stopped feeding on the previous carcass. We conducted three or four feeding experiments per cheetah group (Table 1), with intervals of between 8 and 21 days (mean ± sd: 14.3±4.1 days) between experiments.

**Table 1:** Prey species and prey body mass provided to cheetahs during 15 feeding experiments, prey mass consumed and scats excreted by cheetahs, prey mass consumed per collectable scat and digestibility of prey species.

Prey	Prey provided		Cheetah group		Prey consumed		Scats excreted				Consumed percollectable scat	Digestibility
Species	*n*	Mean kg(*Q*_1_)	Size(*Q*_2_)	ID	Kg	Mean kg per cheetahand prey (*Q*_3_)	*n* collec-table	*n* non-collectable	*n* collectable percheetah and prey (*Q*_4_)	Mean kg of collectableper cheetah and prey (*Q*_6_)	Kg(*Q*_5_ = *Q*_3_/*Q*_4_)	% (*Q*_7_ = (*Q*_3_−*Q*_6_)* 100/*Q*_3_)
Kudu	1	214.00	4	A	28.00	7.00	10	2	2.50	0.162c	2.80	97.69
Gemsbok	1	118.25	4	B	23.25	5.81	11	–	2.75	0.226	2.11	96.12
Gemsbok	1	106.50	4	A	25.00	6.25	9	–	2.25	0.141	2.78	97.75
Kudu	1	106.00	4	B	16.50	4.13	10	–	2.50	0.193	1.65	95.33
Hartebeest	1	98.75	4	B	21.75	5.44	10	–	2.50	0.166	2.18	96.96
Warthog	1	68.50	3	C	17.50	5.83	3	1	1.00	0.121	5.83	97.93
Warthog	1	68.00	4	A	24.00	6.00	8	4	2.00	0.137d	3.00	97.71
Springbok	1	29.25	4	B	21.75	5.44	11	–	2.75	0.324	1.98	94.04
Goat	1	24.00	4	A	13.00	3.25	8	1	2.00	0.188	1.63	94.23
Springbok	1	23.00	3	C	17.00	5.67	10	–	3.33	0.328	1.70	94.22
Springhare	1	3.50	1	D	2.98	2.98	4	–	4.00	0.242	0.75	91.88
Springhare	3	2.83	3	C	8.21	2.74	8	–	2.67	0.340	1.03	87.58
Squirrel	15	0.53	3	C	6.95	0.46b	9	2	0.60	0.061e	0.77	86.90
Squirrel	4	0.50	1	D	1.75	0.44	3	–	0.75	0.043	0.58	90.29
Mouse/Gerbil	14	0.106a	1	D	1.49	0.106a	6	1	0.43	0.022f	0.25	79.06

Correction factors 1 (CF1) and 2 (CF2) were determined by fitting (1) an exponential regression to consumed prey mass per collectable scat (*Q_5_*) as a function of mean prey body mass provided per feeding experiment (*Q_1_*) ( = CF1) and (2) a peak logarithmic normal function to the number of excreted collectable scats (*Q_4_*) as a function of mean prey body mass provided per feeding experiment (*Q_1_*) ( = CF2). For details see text. ^a^ 14 mice and gerbils with a total weight of 1.49 kg were eaten entirely, the remaining 66 with a total weight of 2.74 kg were rejected. Calculations were based on the consumed mice and gerbils. ^b^ each cheetah fed from five subsequently provided squirrels. ^c–f^ mean kg of collectable scats per cheetah are based on mean weights of six, seven, seven and four collectable scats, respectively, multiplied by the number of collectable scats per cheetah.

Following [5], we divided the feeding experiments into three periods: (1) the fasting period before feeding, (2) the feeding day and (3) the fasting period after feeding. During period 1, we removed all cheetah scats each day until no more scats were excreted or until we identified only grass or cheetah hairs in the excrements. Twenty-four hours later, we fed the cheetahs with prey animal(s). Cheetahs fed on the carcasses for between 59 minutes (squirrels) and 3 hours 34 minutes (warthog) with a mean ± sd of 2 hours 40 minutes ±52 minutes (*n* = 13). Approximately ten minutes after the last cheetah of the group stopped feeding, we collected and weighed the remains of the carcass(es). In two feeding experiments with a goat and a springhare the cheetahs were still feeding after 3 hours 25 minutes and 2 hours 30 minutes, respectively, when darkness set in at 19:00 h. In these cases the carcass was left over night and we collected and weighed all remains the next morning. During period 3, we again collected all scats during each day until the cheetahs excreted no more scats or only scats containing grass or cheetah hairs. We distinguished between ‘collectable’ (hard to soft) and ‘non-collectable’ (viscous) scats according to their consistency and included only collectable scats in the determination of correction factors to provide correction factors suitable for field studies [2], [5], [7]–[9]. We counted all collectable and non-collectable scats, and weighed collectable scats spatially separated from non-collectable scats to an accuracy of 2 g. Non-collectable scats covered collectable scats in a few cases, preventing their weighing.

### Data Analysis

We determined CF1 by calculating consumed prey mass per excreted collectable scat (*Q_5_*) for each feeding experiment ( = *Q_3_*/*Q_4_*) and fitting an exponential function of this parameter on mean prey body mass provided per feeding experiment (*Q_1_*) which is a representation of an asymptotic process (Table 1). When we provided more than one carcass, we used the mean of the consumed mass of each carcass. For mice and gerbils we only included those animals that were eaten by the cheetah (*n* = 14, mean weight = 0.106 kg), not those that were rejected and left untouched (*n* = 66, mean weight = 0.042 kg). We determined CF2 by fitting a non-linear regression of the number of excreted collectable scats per cheetah and prey animal (*Q_4_*) on mean prey body mass provided per feeding experiment (*Q_1_*). We initially expected this to also be an asymptotic process, best represented by an exponential function. Alternatively, if at large prey sizes either the behaviour of the predator or the ratio of digestible to indigestible matter changes, then it is possible that the number of excreted collectable scats might decline again. Such a process is usefully represented by a peak logarithmic function. For our study the peak logarithmic function runs best through the data; in the supporting information we also present the exponential function (Fig. S1). For data sets from the literature (see discussion) we use the exponential function; for the Indian wolf (*Canis lupus papllipes*) we also compare the fit of the exponential function with that of the peak logarithmic function (Table S1).

The application of the correction factors to scats collected in the field and the subsequent determination of the diet were based on weights of prey animals consumed by cheetahs. Since identified prey remains in scats rarely provide information on their age and thus approximate weight of the consumed prey individual, age categories of the prey species were estimated and corresponding weights applied [2], [5], [13], [15]. For the hypothetical example, we chose for each prey species the age category most likely to be killed by cheetahs. These were adults of goat, springbok, duiker (*Sylvicapra grimma*), steenbok (*Raphicerus campestris*), springhare and squirrel [24]–[26], juveniles of kudu and hartebeest [27], calves of gemsbok [24], [28] and piglets of warthog [28]. Since cheetahs might also kill other age categories, we discuss how the results will be affected when choosing different age categories and hence prey sizes.

We determined the apparent digestibility for different prey sizes as (mean fresh prey mass consumed per cheetah – mean fresh collectable scat mass per cheetah)*100/mean fresh prey mass consumed per cheetah [15], [29], i.e. (*Q_3_*– *Q_6_*) * 100/*Q_3_*. This digestibility is termed ‘apparent’ because the scats contain also metabolic components from the animal and the fresh scat might also include fluid originating from drinking by the cheetahs [15], [29]. When not all collectable scats could be weighed, we calculated the mean fresh collectable scat mass per cheetah by multiplying the mean weight of the weighed scats by the number of collectable scats (Table 1). We performed all non-parametric tests and regressions using SYSTAT 13.0 (Systat Software Inc.).

## Results

One hundred and twenty (92%) of the 131 scats were collectable scats (Table 1). The ratio of collectable to non-collectable scats when cheetahs were fed with small prey species (springhare, ground squirrel, mouse/gerbil) was similar to that when cheetahs were fed with large prey species (kudu, gemsbok, hartebeest) (Fisher’s exact test, *P* = 0.37, *n* = 85).

### Correction Factor 1

**Figure 2:**
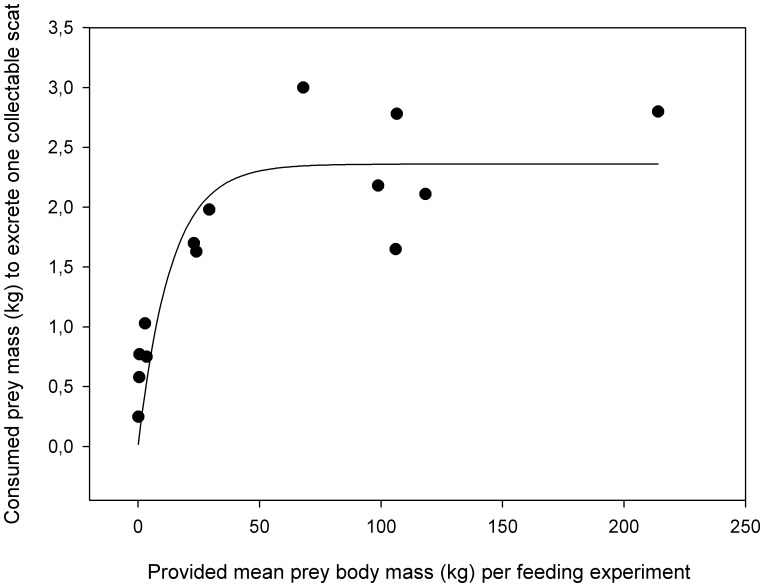
Correction factor 1 (CF1). Consumed mean prey mass (kg) per cheetah to excrete one collectable scat (*Q_5_*) as a function of mean prey body mass (kg) provided per feeding experiment (*Q_1_*). The curve represents CF1 and follows the exponential function *y* = 2.358(1-exp(−0.075*x*). The outlier of the warthog with 5.83 kg prey mass consumed to excrete one collectable scat (Table 1) was excluded from the analysis and figure.

The amount of food from one prey animal a cheetah consumed to excrete one collectable scat (*Q_5_*) increased with prey body mass (*Q_1_*) and levelled out at certain weight of prey body mass. This relationship, the CF1, was described by the exponential function *y* = 2.358(1-exp(−0.075*x*)) with 2.358 kg being the consumed prey mass per collectable scat at which the curve reaches an asymptote (*R^2^* = 0.731, *P*<0.05, *n* = 14) (Fig. 2). The warthog with 5.83 kg eaten per collectable scat (Table 1) was excluded from this regression because its residual (3.07) from the regression line was 3.3 standard deviations from the mean of the residuals of all 15 feeding trials (mean ± sd: 0.62±0.74) and thus regarded as an outlier; its inclusion resulted in a similar equation with a lower fit (*y* = 2.821(1-exp(−0.057*x*)), *R^2^* = 0.493, *P* = ns, *n* = 15).

Apparent digestibility (*y* = *Q_7_*) increased with increasing mean prey body mass per feeding experiment (*x* = *Q_1_*) and levelled out at 94.2% (*y* = 94.192(1-exp(−17.222*x*)), *R^2^* = 0.541, *P*<0.001, *n* = 15).

### Application of Correction Factor 1

The hypothetical example consisted of 100 scats. In these scats, 10 prey species were chosen, each of which was represented in 10 scats. The relative frequency of occurrence for each prey species was 10% (Table 2, column *n*). When applying the empirically determined CF1 to calculate the consumed prey mass per collectable scat (Table 2, column *Q_5_*), the consumed prey mass per species was lowest for small prey species (Table 2, column *n* * *Q_5_*).

**Table 2:** Application of correction factor 1 (CF1, *y* = 2.358(1-exp(−0.075*x*)) with *x* = *Q_1_* and *y* = *Q_5_*) and correction factor 2 (CF2, *y* = 3.094exp(−0.5(ln(*x*/16.370)/2.584)^2^) with *x* = *Q_1_* and *y* = *Q_4_*) derived from the cheetah feeding experiments from this study to a hypothetical example of 100 collectable scats based on a regular sampling scheme, i.e. collected scats were dependent objects.

			*Q*_1_	*n*		*Q*_5_	*n*×*Q*_5_		*Q*_4_	*n*/*Q*_4_		*n*×*Q*_5_/*Q*_1_	
Prey				Scats withprey hair		Consumed mass to excrete 1 scat	Consumed mass to excrete *n* scats		No. of excreted scats per consumed prey	Consumed individuals to excrete *n* scats		Consumed individuals to excrete *n* scats	
										New method		Conventional method	
Species	Age class	Kg		*n*	%	Kg	Kg	%	*n* scats	*n* individuals	%	*n* individuals	%
Kudu	juvenile	100.0^a^		10	10	2.36	23.57	16.04	2.42	4.13	9.88	0.24	2.38
Hartebeest	juvenile	67.5^a^		10	10	2.34	23.43	15.95	2.66	3.76	8.99	0.35	3.51
Goat	adult	43.0^b^		10	10	2.26	22.64	15.41	2.89	3.47	8.29	0.53	5.32
Springbok	adult	39.0^a^		10	10	2.23	22.31	15.19	2.92	3.42	8.18	0.57	5.78
Duiker	adult	18.0^a^		10	10	1.75	17.47	11.89	3.09	3.23	7.74	0.97	9.80
Gemsbok	calf	15.0^a^		10	10	1.59	15.92	10.84	3.09	3.23	7.74	1.06	10.72
Steenbok	adult	11.0^a^		10	10	1.32	13.25	9.02	3.06	3.27	7.82	1.20	12.16
Springhare	adult	3.5^c^		10	10	0.54	5.44	3.71	2.59	3.86	9.24	1.56	15.71
Warthog	piglet	1.2^a^		10	10	0.20	2.03	1.38	1.86	5.39	12.89	1.69	17.08
Squirrel	adult	0.5^c^		10	10	0.09	0.87	0.59	1.24	8.04	19.23	1.74	17.53

Diet composition is presented as frequency of prey occurrence (*n*), consumed mass (*n*×*Q_5_*), and consumed number of prey individuals based on the new method (*n/Q_4_*) and the conventional method (*n*×*Q_5_/Q_1_*). Note the differences in the proportions of the consumed number of prey individuals between the new and the conventional method. For details see text. ^a^ data from [32], ^b^ data from [2], ^c^ data from [33].

### Correction Factor 2

**Figure 3:**
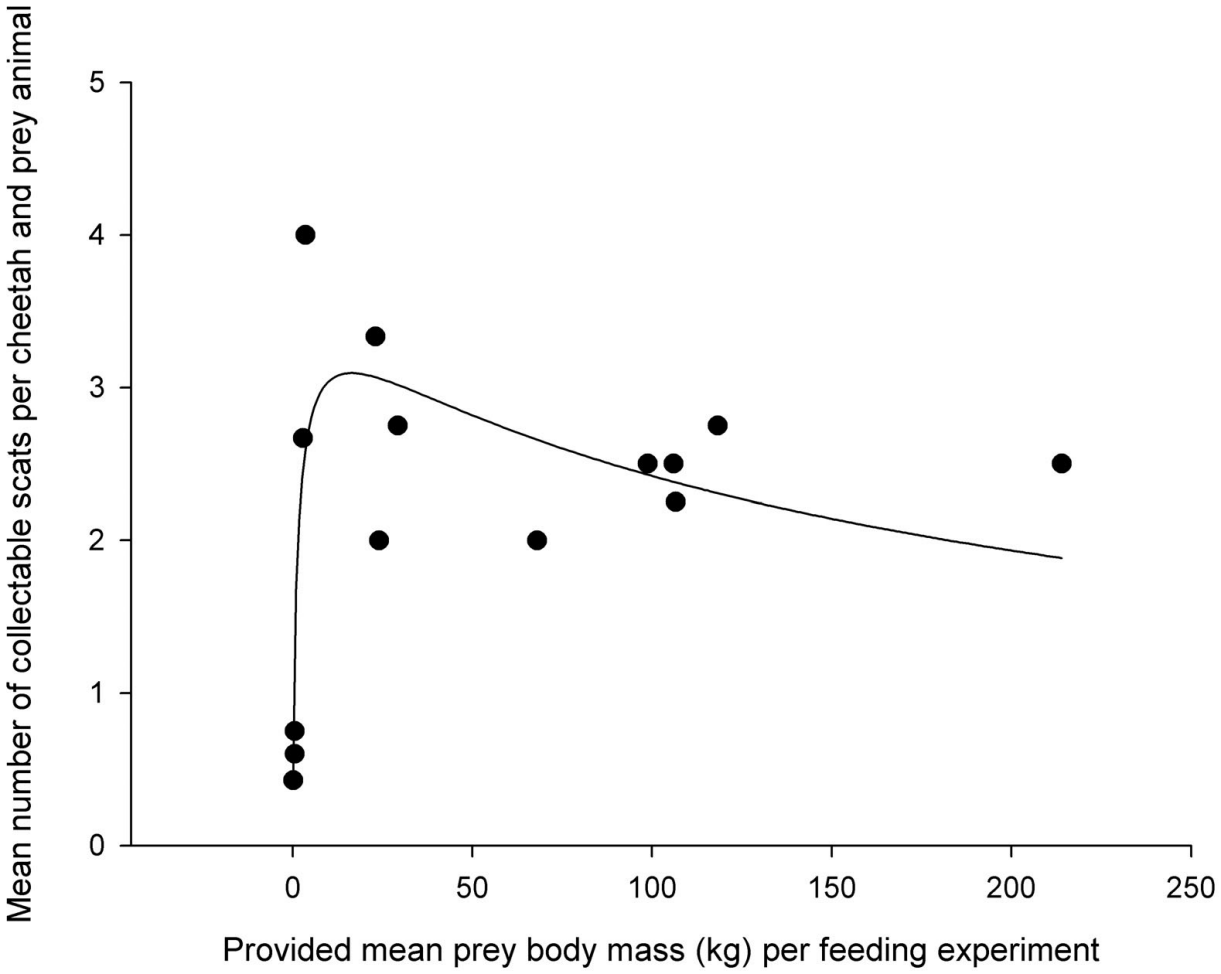
Correction factor 2 (CF2). Mean number of collectable scats excreted per cheetah and prey animal (*Q_4_*) as a function of mean prey body mass (kg) provided per feeding experiment (*Q_1_*). The curve represents CF2 and follows the peak logarithmic normal function *y* = 3.094exp(−0.5(ln(*x*/16.370)/2.584)^2^). The outlier of the warthog with a mean of 1.00 collectable scats per cheetah and prey animal (Table 1) was excluded from the analysis and figure.

The mean number of excreted collectable scats per cheetah and prey animal (*y* = *Q_4_*) increased with mean prey body mass (*x* = *Q_1_*) and then decreased again (Fig. 3). This relationship was described by a peak logarithmic normal function with three parameters (*y* = 3.094exp(-0.5((ln(*x*/16.370))/2.584)^2^), *R^2^* = 0.626, *n* = 14, Fig. 3) with a peak mean number of 3.1 excreted collectable scats per cheetah and prey animal at 16.4 kg of mean prey body mass provided (the first and third coefficient in the equation). Mean mass (kg) of collectable scats per cheetah and prey animal (*y* = *Q_6_*) similarly first increased with the provided mean prey body mass (*x* = *Q_1_*) and then decreased again (*y* = 0.310exp(−0.5((ln(*x*/11.600))/2.022)^2^), *R^2^* = 0.696, *n* = 14), with a peak mean collectable scat weight of 0.31 kg per cheetah and prey animal at 11.60 kg of mean prey body mass provided. The outlier of the warthog with 1 excreted collectable scat per cheetah (Table 1) was excluded from both analyses.

### Sampling Regime and Application of Correction Factor 2

In the hypothetical example, application of CF2 for scats collected by a regular sampling scheme (e.g. daily, weekly, monthly) for solitary cheetahs revealed the highest number of individuals consumed for small prey animals, the lowest number for medium-sized prey animals and a medium number for large prey (Table 2, column *n*/*Q_4_*). This contrasts with the results of the conventional method (dividing consumed mean mass per prey species by the entire prey weight *Q_1_*) where large prey species showed the lowest number in the diet (Table 2, column *n* * *Q_5_/Q_1_*). The numbers of the three prey species with the highest mass in the example, juvenile kudu, juvenile hartebeest and adult goat, were 17.5 times, 10.8 times and 6.6 times higher when using CF2 than the conventional method (Table 2). When scats were collected independently, the number of consumed individuals is *n* (Table 3).

**Table 3:** Determination of the consumed number of prey individuals in a hypothetical example of 100 collectable scats based on CF1 and CF2 derived from the cheetah feeding experiment of this study.

Prey				Consumed number of prey individuals					
		*Q*_1_	*n*	*n*/*Q*_4_/*Q*_2_			*n*		
			Scats with prey hair	Samples collected as dependent objects (more than one scat from each feeding event)			Samples collected independently from each other (one scat per feeding event)		
Species	Age class	Kg	*n*	Solitary	Mean group size		Solitary	Mean group size	
					Two	Four		Two	Four
Kudu	juvenile	100.0^a^	10	4.13	2.07	1.03	10	10	10
Hartebeest	juvenile	67.5^a^	10	3.76	1.88	0.94	10	10	10
Goat	adult	43.0^b^	10	3.47	1.73	0.87	10	10	10
Springbok	adult	39.0^a^	10	3.42	1.71	0.85	10	10	10
Duiker	adult	18.0^a^	10	3.23	1.62	0.81	10	10	10
Gemsbok	calf	15.0^a^	10	3.23	1.62	0.81	10	10	10
Steenbok	adult	11.0^a^	10	3.27	1.64	0.82	10	10	10
Springhare	adult	3.5^c^	10	3.86	1.93	0.97	10	10	10
Warthog	piglet	1.2^a^	10	5.39	2.69	1.35	10	10	10
Squirrel	adult	0.5^c^	10	8.04	4.02	2.01	10	10	10

The calculations are shown for two sampling schemes and three hypothetical mean sizes of feeding groups. *Q_4_* is the number of excreted collectable scats per prey individual and cheetah (see Table 2) and *Q_2_* is feeding group size. For details see text. ^a, b, c^ see Table 2.

## Discussion

Our study demonstrates that carefully correcting for biases inherent in indirect methods of diet determination has a profound effect on the assessment of diet composition and the estimated number of prey animals killed by a carnivore population.

### Correction Factor 1

Our results confirmed that the accuracy of CF1 depends on the number of feeding experiments and the range of prey sizes included. All previous studies that determined such a CF1 fitted a linear regression through their data. However, an exponential function is likely to be biologically more meaningful and physiologically more realistic than a linear function, because it predicts that the amount of prey consumed by a carnivore to excrete one scat reaches an asymptote at large prey sizes (Fig. 2). Such a maximum is reasonable because the total amount of food a carnivore can consume of a large prey is limited and the ratio of indigestible to digestible matter that is consumed does not change after reaching this limit. In line with this, the apparent digestibility increased with increasing prey body mass and reached an upper limit.

Our results show that the consumed prey mass to excrete one collectable scat reaches an asymptotic upper limit at a prey body mass of approximately 50 kg (Fig. 2). Thus, when calculating the diet as consumed prey mass per species there will be little effect on the result whether prey remains of a large prey species in carnivore scats are allocated to the weight of e.g. a heavy adult male, an adult female or a juvenile of the species. This contrasts with the application of a CF1 based on a linear function. If identified prey species are allocated to prey sizes below 50 kg, such as newborns of large prey species or any size class of small species, an accurate assessment of prey body mass becomes important as this will have an impact on the result for CF1 based on both linear and exponential functions.

### Correction Factor 2

#### If scats collected in the field arise from one feeding event (dependent objects)

In this study, we developed a new CF2 that we suggest is applied when carnivore diet is expressed in terms of the proportion of consumed numbers of individuals per prey species and when scats in the field are collected on a regular basis. This correction factor considers that a large carnivore often does not consume the entire prey animal but only part of it [1], [2], [5], [22]. The application of CF2 in a hypothetical example demonstrated that the number of animals per large prey species actually consumed is substantially underestimated by the conventional method (Table 2). This discrepancy increased with increasing prey body mass (Table 2). As with CF1, a precise allocation of prey remains in carnivore scats to age or sex classes is less important for large than for small prey species. This is because CF2 first steeply increases at small to medium prey body masses and subsequently slowly decreases (Fig. 3). The slow decrease of CF2 with increasing prey body mass implies that with increasing body mass a decreasing amount of indigestible prey parts is consumed, resulting in a decreasing number of collectable scats. This might either be a consequence of the carnivore feeding more selectively on carcasses with a large body mass (particularly if feeding group size is small and/or the carnivore does not return to feed repeatedly on the same carcass as is the case in cheetahs [22]), or a change in the characteristics of indigestible body parts such as fur if larger prey individuals have shorter and less dense fur than medium sized prey. The latter would mean that the ratio of indigestible fur to digestible matter decreases with increasing prey size, resulting in a peak at medium sized prey. To our knowledge, there are currently no data on fur characteristics available from prey species from southern Africa to test this idea.

Determination of the diet composition based on the number of consumed individuals per species has two key applications: in ecology it provides evidence on the possible impact of carnivore predators on their prey, and in conservation biology it is important information in the context of human-predator conflicts [2], [13]. If there is little information on age classes of killed prey or if the particular carnivore species in question shows no preference for a particular age class, it seems prudent to determine the diet composition with at least two different prey body mass assessments. This will provide a range of values for the number and proportions of individuals consumed and will make the assessment of the diet robust with respect to both ecological impact and conflict issues.

For very small prey species a particular problem arises. Our results indicate that for such prey animals the consumption of one prey individual will not produce an entire scat (Fig. 3). The smallest prey body mass to produce a complete scat (*y* = *Q_4_*) was 0.337 kg (*x* = *Q_1_*). Cheetah scats with remains of prey with a body mass below this threshold will therefore consist of remains from two consumed individuals of this particular species. Thus, the number of consumed individuals for such prey should be multiplied by a factor of 2.

Scats containing remains of different prey species are likely to change their shape and consistency at different rates, which bias the likelihood of collecting scats containing remains of particular prey species and thus bias diet composition. For example, scats from bobcats (*Lynx rufus*) containing deer remains experienced a larger mass loss than scats containing mice and rats or rabbits when left in the field for three weeks, reducing the likelihood for scats containing deer to be reliably recognised as bobcat scats [30]. The choice of interval between collecting scats should therefore ensure that a scat can be reliably allocated to the carnivore species under study. Some carnivore species use latrines or special marking places that might increase the chance of correctly identifying scats of the studied carnivore. If such a sampling scheme is not feasible, or several similar-sized carnivore species feed on approximately the same prey community in an ecosystem, then allocation of scats to consumer species may require the application of molecular genetic methods to prevent this bias.

#### If scats collected in the field arise from independent feeding events

If scats are collected from carnivores with known individual identity and directly observed during defecation or from immobilised individuals, and if the sampling interval is longer than the time period over which a carnivore excretes scats after having fed from one prey animal, each scat represents a separate feeding event. As a consequence, diet composition estimates differ from the previous sampling scheme by *Q_4_* and for carnivores feeding in groups additionally by the factor *Q_2_* (Table 3). It is therefore important to adjust data analysis by sampling regime and the average feeding group size of the carnivore.

### More than One Prey Species per Scat

Our hypothetical example is based on scats containing remains of one prey species only. However, scats sometimes contain more than one prey species. Studies that determined the dry mass or volume of all prey remains in the scats use the proportions of dry masses or volumes of the different species in the scats, sum these proportions up and apply CF1 to determine the consumed mass per prey species applied [5], [10], [18], [31]. If no information on dry mass or volume is available, we suggest to allocate equal *Q_5_* contributions of the different prey species to the scats, as this is likely to represent a mean *Q_5_* contribution to the scats in a reasonably large sample size. For example, if 10 scats contain remains of prey species A and prey species B, the consumed mass per prey species in a solitary carnivore should be calculated as (10×*Q_5A_*×0.5) + (10×*Q_5B_*×0.5). Similarly, to calculate the consumed number of individuals per prey species we suggest to allocate equal *Q_4_* contributions of different prey species to the scats. Using the same example, the consumed number of individuals per prey species would be (10/*Q_4A_*×0.5) + (10/*Q_4B_*×0.5).

### Determination of CF1 and CF2 from Other Studies

Feeding experiments have been conducted for other carnivores such as the wolf [7]–[9], cougar [6] and Eurasian lynx [16]. All these studies recorded the prey mass of the prey animals provided, the prey mass consumed by the carnivores, the number of scats produced by the carnivores and the number of carnivores feeding from the prey animals. With this information CF1 and CF2 can be derived in retrospect and used for future diet determinations without the need to repeat the feeding experiments. We reanalysed published raw data of feeding experiments with wolves in North America [8], Europe [9] and India [7], and with Eurasian lynxes [16] by following the steps presented in Table 1 to determine CF1 and CF2 (Table S1). For all four studies, we fitted an exponential function to derive CF1 (wolf in North America: *y* = 1.798(1-exp(−0.008*x*); in Europe: *y* = 0.621(1-exp(−0.012*x*); in India: *y* = 1.382(1-exp(-0.020*x*); lynx: *y* = 1.045(1-exp(−0.145*x*)). We also fitted an exponential function through the data from the four studies to derive CF2 (wolf in North America: *y* = 37.311(1-exp(−0.021*x*); in Europe: *y* = 39.473(1-exp(−0.061*x*); in India: *y* = 13.940(1-exp(−0.980*x*); Eurasian lynx: *y* = 6.995(1-exp(−0.201*x*)), however, the data from the study on wolves in India were best described by a peak logarithmic normal function with three parameters (*y* = 21.792exp(−0.5((ln(*x*/6.444))/1.273)^2^)), similar to our cheetah study (calculations and figures in Table S1).

Our results for CF2 suggest that the appropriate function to derive CF2 differs between carnivores living in temperate and tropical areas. Whereas CF2 reaches an asymptote in temperate areas, CF2 in India and Namibia first steeply increased at small to medium prey body masses and subsequently decreased slowly (Fig. 3 and Table S1). Either large prey animals in tropical areas have shorter or less dense fur than medium-sized prey animals whereas in temperate areas this is not the case, or tropical carnivores feed more selectively from larger prey, feed in smaller groups or are less likely to return to a carcass than temperate carnivores.

### Application of CF1 and CF2 to Other Studies

The application of CF1 and CF2 for cheetahs established by our study to another study on cheetah diet in Namibia based on scat analyses [2] revealed, as expected from the hypothetical example in Table 2, a higher number of larger and a lower number of smaller prey animals consumed than previously estimated (Table S2). The new method indicated that 9.3% and 12.1% of the prey individuals consumed by cheetahs in [2]’s study were livestock (cattle and sheep) and hares, respectively, whereas the conventional method suggested 3.7% and 40.4%. Thus, cheetahs had a 2.5 times higher proportion of livestock animals and a 3.3 times lower proportion of hares in their diet than previously thought. Underestimation of predator-human conflict on the basis of inaccurate consumption data is likely to reduce the efficiency of management measures by farmers to reduce the conflict or of conservation measures to mitigate such conflicts.

As far as we are aware, feeding experiments have not yet been conducted for carnivores such as the tiger (*Panthera tigris*), leopard (*Panthera pardus*) or dhole (*Cuon alpinus*) in India. We determined the diet of these carnivore species in terms of consumed prey mass and number of prey individuals (Table S2) by applying CF1 and CF2 derived from our cheetah feeding experiments for tiger and leopard and from feeding experiments with Indian wolves (Table S1) for the dhole. Compared to estimates based on the conventional method [13], the number of larger prey was higher and the number of smaller prey lower, predator pressure on gaur (*Bos gaurus*) and sambar (*Cervus unicolor*) was between 1.7 and 2.2 times higher, and chital (*Axis axis*) was the main prey only for leopard and dhole, overriding previous assessment of niche overlap and predator-prey relationships (Table S2).

With all the reservations that apply when approximating the diet of one predator with correction factors derived from another predator, we still consider this a worthwhile approach. In essence, this implies that the error introduced by transferring estimates of correction factors between species is less than leaving the two correction factors out altogether. For instance, the peak number of collectable scats per individual wolf and prey animal was 21.8 (India) and 37.3 (North America) and 39.5 (Europe) (Table S1); similar peak numbers for lynx in Europe was 7.0 (Table S1) and for the cheetahs in this study 3.1. If applied within the same geographical area and taxon, these differences are less substantial than the corrections that resulted from comparing the cheetah studies (2.5 and 3.3 fold changes in consumed numbers of livestock animals and hares). Hence, if no CFs are available for the studied species we suggest to apply (1) the cheetah CF1 and CF2 derived in this study to Felidae of similar or larger size in hot and tropical areas, (2) the wolf CF1 and CF2 derived from studies in North America, Europe and India to Canidae from the respective geographical areas and (3) the Eurasian lynx CF1 and CF2 to Felidae of similar size in temperate areas. Such approximations also need to consider the actual mean sizes of feeding groups of the species under study and correctly identify the sampling scheme used to collect scats (Table 3).

CF1 and CF2 determined in this study are only applicable to carnivores feeding chiefly on mammalian prey species and on species that are not completely consumed. For carnivore species mainly feeding on fruits, invertebrates, birds and small mammals such as black-backed jackals, side-striped jackals and red fox, the diet should be determined using the specific conversion factors for these food items [18], [19], [20], [21] and following the guidelines in [20]. If small mammals are not completely consumed, CF2 can be derived from the mammalian data of such feeding experiments following the new method. For felids and canids in geographical areas not covered by the CF1s and CF2s presented and suggested here, new feeding experiments should be conducted.

### Feeding Experiments

The design of the feeding experiments will influence the outcome in terms of the non-linear functions for CF1 and CF2. How much and which parts of a prey animal a carnivore consumes depends on factors such as its state of hunger, the prey mass provided and competition with conspecifics [15], [22]. Therefore, if CF1 and CF2 are applied to scats collected in the field, feeding experiments should simulate as closely as possible natural feeding situations (see Table S3). Although it might not be possible to completely simulate the situation under free-ranging conditions, the determination of CF2 is desirable because even a rough approximation of this factor will considerably reduce the bias in the number of consumed prey species produced by the conventional method.

## Supporting Information

Figure S1 C**orrection factor 2 (CF2) described as exponential function from our data set in** **table 1** **.** Mean number of collectable scats excreted per cheetah and prey animal (*Q_4_*) as a function of mean prey body mass (kg) provided per feeding experiment (*Q_1_*). CF2 follows the exponential function *y* = 2.654(1-exp(−0.960*x*)), *R^2^* = 0.705, *P*<0.05, *n* = 14. For details and comparison with CF2 described as a peak logarithmic function see Figure 3.
(TIF)

Table S1 **Determination of correction factors 1 (CF1) and 2 (CF2) from four published feeding experiments with the new method.** We used the studies on wolves from North America [8], Europe [9] and India [7] and Eurasian lynx from Europe [16]. For each study we present in a table the published data and our calculations to derive CF1 and CF2, and the figures with the regression curves and the equations for CF1 and CF2.
(DOC)

Table S2 **Application of correction factors 1 (CF1) and 2 (CF2) to four published studies on carnivore diet following the new method.** We applied CF1 and CF2 derived from our feeding experiments with cheetahs (*Acinonyx jubatus*) to a cheetah study in Namibia [2], and a tiger (*Panthera tigris*) and leopard (*P. pardus*) study in India [13], and CF1 and CF2 derived in this study from a feeding experiment with Indian wolves [7] (see Table S1) to a dhole (*Cuon alpinus*) study in India [13].
(DOC)

Table S3 **Guidelines for feeding experiments to ensure the experiments simulate as closely as possible natural feeding situations.**
(DOC)
