# Supplementary material for: An Advanced Method to Assess the Diet of Free-Ranging Large Carnivores Based on Scats
Source: PLoS One. 2012 Jun 8;7(6):e38066. doi: 10.1371/journal.pone.0038066 (PMC3371055; doi:10.1371/journal.pone.0038066)
Supplement: Table S1 — Determination of correction factors 1 (CF1) and 2 (CF2) from four published feeding experiments with the new method. We used the studies on wolves from North America [8], Europe [9] and India [7] and Eurasian lynx from Europe [16]. For each study we present in a table the published data and our calculations to derive CF1 and CF2, and the figures with the regression curves and the equations for CF1 and CF2. (DOC) [file pone.0038066.s002.doc]

**Table S1.** **Determination of correction factors 1 (CF1) and 2 (CF2) from four published feeding experiments with the new method.** We used the studies on wolves from North America [1], Europe [2] and India [3] and Eurasian lynx from Europe [4]. For each study we present in a table the published data and our calculations to derive CF1 and CF2, and the figures with the regression curves and the equations for CF1 and CF2.

**Wolves from North America.** Prey species and prey body mass provided to three wolves (*Canis lupus*) in nine feeding experiments conducted by [1], prey mass consumed and scats excreted by wolves, and prey mass consumed per collectable scat. Prey species were mule deer (*Odocoileus hemionus*), red deer (*Cervus elaphus*) and moose (*Alces alces*). Correction factors were determined by fitting exponential regressions to (1) consumed prey mass per collectable scat (*Q_5_*) as a function of mean prey body mass provided per feeding experiment (*Q_1_*) (=CF1) and (2) the number of collectable scats excreted per wolf and prey animal (*Q_4_*) as a function of mean prey body mass provided per feeding experiment (*Q_1_*) (=CF2).

| Prey |  | Prey provided | |  | Wolf group |  | Prey consumed | |  | Scats excreted | |  | Consumed per collectable scat |
| --- | --- | --- | --- | --- | --- | --- | --- | --- | --- | --- | --- | --- | --- |
| Species^a^ |  | *n*^a^ | Mean kg (*Q_1_*)^a^ |  | Size (*Q_2_*)^a^ |  | Kg^a^ | Mean kg per wolf |  | *n* collectable^a^ | *n* collectable per |  | Kg (*Q_5_ = Q_3_* / *Q_4_*) |
|  |  |  |  |  |  |  |  | and prey (*Q_3_*) |  |  | wolf and prey (*Q_4_*) |  |  |
| Mule deer |  | 1 | 32.70 |  | 3 |  | 30.90 | 10.30 |  | 72 | 24.00 |  | 0.43 |
| Mule deer |  | 1 | 41.80 |  | 3 |  | 37.70 | 12.57 |  | 61 | 20.33 |  | 0.62 |
| Mule deer |  | 1 | 50.00 |  | 3 |  | 41.80 | 13.93 |  | 62 | 20.67 |  | 0.67 |
| Red deer |  | 1 | 60.90 |  | 3 |  | 52.70 | 17.57 |  | 99 | 33.00 |  | 0.53 |
| Red deer |  | 1 | 104.50 |  | 3 |  | 73.60 | 24.53 |  | 71 | 23.67 |  | 1.04 |
| Moose |  | 1 | 114.50 |  | 3 |  | 107.20 | 35.73 |  | 93 | 31.00 |  | 1.15 |
| Moose |  | 1 | 157.30 |  | 3 |  | 142.80 | 47.60 |  | 110 | 36.67 |  | 1.30 |
| Red deer |  | 1 | 239.10 |  | 3 |  | 230.50 | 76.83 |  | 130 | 43.33 |  | 1.77 |
| Moose |  | 1 | 327.10 |  | 3 |  | 168.20 | 56.07 |  | 109 | 36.33 |  | 1.54 |

^a^ data from [1]

Consumed mean prey mass (kg) per wolf to excrete one collectable scat (*Q_5_*) as a function of mean prey body mass (kg) provided per feeding experiment (*Q_1_*). Data are from [1]. The curve represents correction factor 1 (CF1), *y* = 1.798(1-exp(-0.008*x*)), *R^2^* = 0.937, *P* < 0.001 , *n* = 9.

Mean number of collectable scats excreted per wolf and prey animal (*Q_4_*) as a function of mean prey body mass (kg) provided per feeding experiment (*Q_1_*). Data from [1]. The curve represents correction factor 2 (CF2): *y* = 37.311(1-exp(-0.021*x*)), *R^2^* = 0.583, *P* < 0.01 , *n* = 9.

**Wolves from Europe.** Prey species and prey body mass provided to five wolves (*Canis lupus*) in ten feeding experiments conducted by [2], prey mass consumed and scats excreted by wolves, and prey mass consumed per collectable scat. Prey species were roe deer (*Capreolus capreolus*), red deer (*Cervus elaphus*) and wild boar (*Sus scrofa*). Correction factors were determined by fitting exponential regressions to (1) consumed prey mass per collectable scat (*Q_5_*) as a function of mean prey body mass provided per feeding experiment (*Q_1_*) (=CF1) and (2) the number of collectable scats excreted per wolf and prey animal (*Q_4_*) as a function of mean prey body mass provided per feeding experiment (*Q_1_*) (=CF2).

| Prey |  | Prey provided | |  | Wolf group |  | Prey consumed | |  | Scats excreted | |  | Consumed per collectable scat |
| --- | --- | --- | --- | --- | --- | --- | --- | --- | --- | --- | --- | --- | --- |
| Species^a^ |  | *n*^a^ | Mean kg (*Q_1_*)^a^ |  | Size (*Q_2_*)^a^ |  | Kg^a^ | Mean kg per wolf |  | *n* collectable^a^ | *n* collectable per |  | Kg (*Q_5_ = Q_3_* / *Q_4_*) |
|  |  |  |  |  |  |  |  | and prey (*Q_3_*) |  |  | wolf and prey (*Q_4_*) |  |  |
| Roe deer |  | 1 | 15.30 |  | 5 |  | 14.50 | 2.90 |  | 166 | 33.20 |  | 0.09 |
| Roe deer |  | 1 | 19.80 |  | 5 |  | 19.50 | 3.90 |  | 154 | 30.80 |  | 0.13 |
| Roe deer |  | 1 | 23.80 |  | 5 |  | 23.00 | 4.60 |  | 144 | 28.80 |  | 0.16 |
| Red deer |  | 1 | 29.00 |  | 5 |  | 26.80 | 5.36 |  | 104 | 20.80 |  | 0.26 |
| Wild boar |  | 1 | 33.90 |  | 5 |  | 32.20 | 6.44 |  | 176 | 35.20 |  | 0.18 |
| Wild boar |  | 1 | 42.50 |  | 5 |  | 42.00 | 8.40 |  | 154 | 30.80 |  | 0.27 |
| Wild boar |  | 1 | 51.00 |  | 5 |  | 50.70 | 10.14 |  | 198 | 39.60 |  | 0.26 |
| Red deer |  | 1 | 82.00 |  | 5 |  | 78.70 | 15.74 |  | 208 | 41.60 |  | 0.38 |
| Red deer |  | 1 | 116.00 |  | 5 |  | 106.30 | 21.26 |  | 249 | 49.80 |  | 0.43 |
| Wild boar |  | 1 | 118.50 |  | 5 |  | 85.80 | 17.16 |  | 162 | 32.40 |  | 0.53 |

^a^ data from [2]

Consumed mean prey mass (kg) per wolf to excrete one collectable scat (*Q_5_*) as a function of mean prey body mass (kg) provided per feeding experiment (*Q_1_*). Data from [2]. The curve represents correction factor 1 (CF1), *y* = 0.621(1-exp(-0.012*x*)), *R^2^* = 0.927, *P* < 0.01 , *n* = 10.

Mean number of collectable scats excreted per wolf and prey animal (*Q_4_*) as a function of mean prey body mass (kg) provided per feeding experiment (*Q_1_*). Data from [2]. The curve represents correction factor 2 (CF2), *y* = 39.473(1-exp(-0.061*x*)), *R^2^* = 0.221, *P* < 0.05 , *n* = 10.

**Wolves from India.** Prey species and prey body mass provided to one to five Indian wolves (*Canis lupus papllipes*) in 12 feeding experiments conducted by [3], prey mass consumed and scats excreted by wolves, and prey mass consumed per collectable scat. Prey species were rat (*Rattus norvegicus*), rabbit (*Oryctolagus spp.*), goat (*Capra hircus*), sheep (*Ovis aries*) and buffalo (*Bubalus bubalus*). Correction factors were determined by fitting (1) an exponential regression to consumed prey mass per collectable scat (*Q_5_*) as a function of mean prey body mass provided per feeding experiment (*Q_1_*) (=CF1) and (2) a peak logarithmic normal function as well as an exponential regression to the number of collectable scats excreted per wolf and prey animal (*Q_4_*) as a function of mean prey body mass provided per feeding experiment (*Q_1_*) (=CF2).

| Prey |  | Prey provided | |  | Wolf group |  | Prey consumed | |  | Scats excreted | |  | Consumed per collectable scat |
| --- | --- | --- | --- | --- | --- | --- | --- | --- | --- | --- | --- | --- | --- |
| Species^a^ |  | *n*^a^ | Mean kg (*Q_1_*)^a^ |  | Size (*Q_2_*)^a^ |  | Kg^a^ | Mean kg per wolf |  | *n* collectable^a^ | *n* collectable per |  | Kg (*Q_5_ = Q_3_* / *Q_4_*) |
|  |  |  |  |  |  |  |  | and prey (*Q_3_*) |  |  | wolf and prey (*Q_4_*) |  |  |
| Rat |  | 1 | 0.80 |  | 1 |  | 0.80 | 0.80 |  | 8 | 8.00 |  | 0.10 |
| Rabbit |  | 1 | 1.50 |  | 1 |  | 1.40 | 1.40 |  | 10 | 10.00 |  | 0.14 |
| Rabbit |  | 1 | 1.50 |  | 1 |  | 1.50 | 1.50 |  | 10 | 10.00 |  | 0.15 |
| Rabbit |  | 1 | 1.50 |  | 1 |  | 1.40 | 1.40 |  | 11 | 11.00 |  | 0.13 |
| Goat kid |  | 1 | 5.50 |  | 1 |  | 5.50 | 5.50 |  | 21 | 21.00 |  | 0.26 |
| Sheep lamb |  | 1 | 7.00 |  | 1 |  | 6.24 | 6.24 |  | 25 | 25.00 |  | 0.25 |
| Goat |  | 1 | 19.00 |  | 4 |  | 17.76 | 4.44 |  | 71 | 17.75 |  | 0.25 |
| Sheep |  | 1 | 23.00 |  | 3 |  | 17.54 | 5.85 |  | 33 | 11.00 |  | 0.53 |
| Goat |  | 1 | 25.50 |  | 3 |  | 20.04 | 6.68 |  | 29 | 9.67 |  | 0.69 |
| Goat |  | 1 | 26.50 |  | 5 |  | 16.26 | 3.25 |  | 23 | 4.60 |  | 0.71 |
| Buffalo calf |  | 1 | 35.00 |  | 4 |  | 27.30 | 6.83 |  | 58 | 14.50 |  | 0.47 |
| Juvenile cattle |  | 1 | 65.00 |  | 3 |  | 27.83 | 9.28 |  | 26 | 8.67 |  | 1.07 |

^a^ data from [3]

Consumed mean prey mass (kg) per wolf to excrete one collectable scat (*Q_5_*) as a function of mean prey body mass (kg) provided per feeding experiment (*Q_1_*). Data from [3]. The curve represents correction factor 1 (CF1), *y* = 1.382(1-exp(-0.021*x*)), *R^2^* = 0.820, *P* = ns, *n* = 12.

Mean number of collectable scats excreted per wolf and prey animal (*Q_4_*) as a function of mean prey body mass (kg) provided per feeding experiment (*Q_1_*). Data from [3]. The curve represents correction factor 2 (CF2) as a peak logarithmic normal function, *y* = 21.792exp(-0.5((ln(*x*/6.444))/1.273)^2^), *R^2^* = 0.637, *n* = 12.

Mean number of collectable scats excreted per wolf and prey animal (*Q_4_*) as a function of mean prey body mass (kg) provided per feeding experiment (*Q_1_*). Data from [3]. The curve represents correction factor 2 (CF2) as an exponential regression, *y* = 13.940(1-exp(-0.980*x*)), *R^2^* = 0.133, *n* = 12.

**Eurasian lynx from Europe.** Prey species and prey body mass provided to two lynxes (*Lynx lynx*) in nine feeding experiments conducted by [4], prey mass consumed and scats excreted by lynxes, and prey mass consumed per collectable scat. Prey species were mice (*Mus musculus*), European hare (*Lepus europaeus*), wild boar (Sus scrofa), roe deer (*Capreolus capreolus*), fallow deer (*Dama dama*) and moufflon (*Ovis ammon musimon*). Correction factors were determined by fitting exponential regressions to (1) consumed prey mass per collectable scat (*Q_5_*) as a function of mean prey body mass provided per feeding experiment (*Q_1_*) (=CF1) and (2) number of collectable scats excreted per wolf and prey animal (*Q_4_*) as a function of mean prey body mass provided per feeding experiment (*Q_1_*) (=CF2).

| Prey |  | Prey provided | |  | Lynx group |  | Prey consumed | |  | Scats excreted | |  | Consumed per collectable scat |
| --- | --- | --- | --- | --- | --- | --- | --- | --- | --- | --- | --- | --- | --- |
| Species^a^ |  | *n*^a^ | Mean kg (*Q_1_*)^a^ |  | Size (*Q_2_*)^a^ |  | Kg^a^ | Mean kg per lynx |  | *n* collectable^a^ | *n* collectable per |  | Kg (*Q_5_ = Q_3_* / *Q_4_*) |
|  |  |  |  |  |  |  |  | and prey (*Q_3_*) |  |  | lynx and prey (*Q_4_*) |  |  |
| Mice |  | 123 | 0.03 |  | 2 |  | 0.03 | 0.02 |  | 9 | 0.07 |  | 0.22 |
| European hare |  | 1 | 4.70 |  | 2 |  | 4.60 | 2.30 |  | 6 | 3.00 |  | 0.77 |
| Wild boar |  | 1 | 8.60 |  | 2 |  | 7.40 | 3.70 |  | 15 | 7.50 |  | 0.49 |
| Wild boar |  | 1 | 13.00 |  | 2 |  | 10.90 | 5.45 |  | 14 | 7.00 |  | 0.78 |
| Roe deer |  | 1 | 15.50 |  | 2 |  | 11.20 | 5.60 |  | 11 | 5.50 |  | 1.02 |
| Roe deer |  | 1 | 17.50 |  | 2 |  | 12.60 | 6.30 |  | 15 | 7.50 |  | 0.84 |
| Roe deer |  | 1 | 20.50 |  | 2 |  | 14.50 | 7.25 |  | 11 | 5.50 |  | 1.32 |
| Fallow deer |  | 1 | 21.40 |  | 2 |  | 13.60 | 6.80 |  | 13 | 6.50 |  | 1.05 |
| Moufflon |  | 1 | 25.10 |  | 2 |  | 13.50 | 6.75 |  | 16 | 8.00 |  | 0.84 |

^a^ data from [4]

Consumed mean prey mass (kg) per lynx to excrete one collectable scat (*Q_5_*) as a function of mean prey body mass (kg) provided per feeding experiment (*Q_1_*). Data from [4]. The curve represents correction factor 1 (CF1), *y* = 1.045(1-exp(-0.145*x*)), *R^2^* = 0.731, *P* = ns, *n* = 9.

Mean number of collectable scats excreted per lynx and prey animal (*Q_4_*) as a function of mean prey body mass (kg) provided per feeding experiment (*Q_1_*) and. Data from [4]. The curve represents correction factor 2 (CF2), *y* = 6.995(1-exp(-0.201*x*)), *R^2^* = 0.811, *P* = ns, *n* = 9.

**References**

1. Weaver JL (1993) Refining the equation for interpreting prey occurrence in gray wolf scats. J Wildl Manage 57: 534-538.

2. Ruehe F, Buschmann I, Wameling A (2003) Two models of assessing the prey mass of European ungulates from wolf scats. Acta Theriol 48: 527-537.

3. Jethva BD, Jhala YV (2004) Computing biomass consumption from prey occurences in Indian wolf scats. Zoo Biol 23: 513-520.

4. Rühe F, Burmester T, Ksinsik M (2007) Data for estimating eaten prey masses from Eurasian lynx Lynx lynx scats in Central and East Europe. Acta Theriol 52: 317-322.
