# Supplementary material for: An Advanced Method to Assess the Diet of Free-Ranging Large Carnivores Based on Scats
Source: PLoS One. 2012 Jun 8;7(6):e38066. doi: 10.1371/journal.pone.0038066 (PMC3371055; doi:10.1371/journal.pone.0038066)
Supplement: Table S2 — Application of correction factors 1 (CF1) and 2 (CF2) to four published studies on carnivore diet following the new method. We applied CF1 and CF2 derived from our feeding experiments with cheetahs (Acinonyx jubatus) to a cheetah study in Namibia [2], and a tiger (Panthera tigris) and leopard (P. pardus) study in India [13], and CF1 and CF2 derived in this study from a feeding experiment with Indian wolves [7] (see Table S1) to a dhole (Cuon alpinus) study in India [13]. (DOC) [file pone.0038066.s003.doc]

**Table S2. Application of correction factors 1 (CF1) and 2 (CF2) to four published studies on carnivore diet following the new method.** We applied CF1 and CF2 derived from our feeding experiments with cheetahs (*Acinonyx jubatus*) to a cheetah study in Namibia [1], and a tiger (*Panthera tigris*) and leopard (*P. pardus*) study in India [2], and CF1 and CF2 derived in this study from a feeding experiment with Indian wolves [3] (see Table S1) to a dhole (*Cuon alpinus*) study in India [2].

**CF1 and CF2 of cheetahs applied to cheetah scats.** Application of CF1 and CF2 derived from the cheetah feeding experiment presented in this study to 33 cheetah scats collected in the field by [1]. [1] used for their diet determination the conventional method with one CF based on the linear relationship *y* = 0.0098*x*+0.3425 with *x* being the mean prey body mass provided per feeding experiment and *y* the consumed mean prey mass to excrete one collectable scat. Consumed prey mass to excrete one scat and the diet composition based on consumed mass and consumed number of prey individuals, respectively, were calculated with the new method and the conventional method. Cheetah feeding group size was considered to be one and samples were considered to be collected dependently from each other.

| Prey | | |  | Scats with prey hair |  | Consumed mass to excrete 1 scat (kg) | |  | Diet composition based on consumed mass (%) | |  | Diet composition based on consumed individuals (%) | |
| --- | --- | --- | --- | --- | --- | --- | --- | --- | --- | --- | --- | --- | --- |
| Species^a^ | Assumed age class^a^ | Assumed weight (kg) ^a^ |  | *n*^a^ |  | New method | [1]^a^ |  | New  method | [1]^b^ |  | New  method | [1]^b^ |
| Sheep (*Ovis aries*) | adult | 59.00 |  | 1 |  | 2.33 | 0.92 |  | 4.22 | 5.01 |  | 3.24 | 1.11 |
| Warthog (*Phacochoerus africanus*) | adult | 45.00 |  | 1 |  | 2.28 | 0.78 |  | 4.12 | 4.26 |  | 3.09 | 1.23 |
| Cattle | calf | 40.00 |  | 2 |  | 2.24 | 0.73 |  | 8.11 | 8.00 |  | 6.07 | 2.60 |
| Eland (*Taurotragus oryx*) | calf | 36.00 |  | 7 |  | 2.20 | 0.70 |  | 27.88 | 26.49 |  | 20.98 | 9.58 |
| Kudu (*Tragelaphus strepsiceros*) | calf | 16.00 |  | 10 |  | 1.65 | 0.50 |  | 29.84 | 27.18 |  | 28.61 | 22.10 |
| Hartebeest (*Alcelaphus buselaphus*) | calf | 15.00 |  | 3 |  | 1.59 | 0.49 |  | 8.65 | 7.99 |  | 8.59 | 6.93 |
| Gemsbok (*Oryx gazelle*) | calf | 15.00 |  | 2 |  | 1.59 | 0.49 |  | 5.77 | 5.33 |  | 5.72 | 4.62 |
| Steenbok (*Raphicerus campestris*) | adult | 11.20 |  | 4 |  | 1.34 | 0.45 |  | 9.71 | 9.85 |  | 11.57 | 11.44 |
| Hare | adult | 1.90 |  | 3 |  | 0.31 | 0.36 |  | 1.70 | 5.90 |  | 12.14 | 40.39 |

^a^ data from [1]

^b^ data derived [1]

**CF1 and CF2 of cheetahs applied to tiger scats.** Application of CF1 and CF2 derived from the cheetah feeding experiment presented in this study to 422 tiger scats collected in the field by [2]. [2] used for their diet determination the conventional method with one CF based on the linear relationship *y* = 0.035*x*+1.980 derived from a cougar feeding experiment [4] with *x* being the mean prey body mass provided per feeding experiment and *y* the consumed mean prey mass to excrete one collectable scat. Consumed prey mass to excrete one scat and the diet composition based on consumed mass and consumed number of prey individuals, respectively, were calculated with the new method and the conventional method. Tiger feeding group size was considered to be one and samples were considered to be collected dependently from each other.

| Prey |  |  | Scats with prey hair | |  | | Consumed mass to excrete 1 scat (kg) | |  | | Diet composition based on consumed mass (%) | |  | | Diet composition based on consumed individuals (%) | |
| --- | --- | --- | --- | --- | --- | --- | --- | --- | --- | --- | --- | --- | --- | --- | --- | --- |
| Species^a^ | Assumed weight (kg) ^a^ |  | *n*^a^ |  | | New method | | [2]^a^ |  | New  method | | [2]^a^ |  | New  method | | [2]^a^ |
| Gaur (*Bos gaurus*) | 287.00 |  | 106 |  | | 2.36 | | 12.03 |  | 26.23 | | 42.74 |  | 32.94 | | 14.71 |
| Sambar (*Cervus unicolor*) | 212.00 |  | 99 |  | | 2.36 | | 9.40 |  | 24.50 | | 31.21 |  | 27.20 | | 14.54 |
| Chital (*Axis axis*) | 55.00 |  | 145 |  | | 2.32 | | 3.91 |  | 35.30 | | 18.99 |  | 27.21 | | 34.10 |
| Wild pig (Sus scrofa) | 38.00 |  | 40 |  | | 2.22 | | 3.31 |  | 9.33 | | 4.44 |  | 7.09 | | 11.54 |
| Muntjac (*Muntiacus muntjak*) | 20.00 |  | 11 |  | | 1.83 | | 2.68 |  | 2.11 | | 0.99 |  | 1.85 | | 4.88 |
| Four-horned antelope (*Tetracerus quadricornis*) | 19.00 |  | 5 |  | | 1.79 | | 2.65 |  | 0.94 | | 0.44 |  | 0.84 | | 2.31 |
| Langur (*Semnopithecus entellus*) | 8.00 |  | 10 |  | | 1.06 | | 2.26 |  | 1.12 | | 0.76 |  | 1.75 | | 9.36 |
| Chevrotain (*Tragulus meminna*) | 5.00 |  | 6 |  | | 0.74 | | 2.16 |  | 0.46 | | 0.43 |  | 1.12 | | 8.57 |

^a^ data from [2]

**CF1 and CF2 of cheetahs applied to leopard scats.** Application of CF1 and CF2 derived from the cheetah feeding experiment presented in this study to 111 leopard scats collected in the field by [2]. [2] used for their diet determination the conventional method with one CF based on the linear relationship *y* = 0.035*x*+1.980 derived from a cougar feeding experiment [4] with *x* being the mean prey body mass provided per feeding experiment and *y* the consumed mean prey mass to excrete one collectable scat. Consumed prey mass to excrete one scat and the diet composition based on consumed mass and consumed number of prey individuals, respectively, were calculated with the new method and the conventional method. Leopard feeding group size was considered to be one and samples were considered to be collected dependently from each other.

| Prey |  |  | Scats with prey hair |  | Consumed mass to excrete 1 scat (kg) | |  | Diet composition based on consumed mass (%) | |  | Diet composition based on consumed individuals (%) | |
| --- | --- | --- | --- | --- | --- | --- | --- | --- | --- | --- | --- | --- |
| Species^a^ | Assumed weight (kg) ^a^ |  | *n*^a^ |  | New method | [2]^a^ |  | New  method | [2]^a^ |  | New  method | [2]^a^ |
| Gaur | 85.00 |  | 12 |  | 2.35 | 4.96 |  | 12.08 | 15.03 |  | 12.10 | 5.90 |
| Sambar | 62.00 |  | 8 |  | 2.34 | 4.15 |  | 7.99 | 8.39 |  | 7.52 | 4.51 |
| Chital | 48.00 |  | 60 |  | 2.29 | 3.66 |  | 58.83 | 55.50 |  | 53.84 | 38.57 |
| Wild pig | 37.00 |  | 11 |  | 2.21 | 3.28 |  | 10.40 | 9.11 |  | 9.51 | 8.21 |
| Muntjac | 20.00 |  | 2 |  | 1.83 | 2.68 |  | 1.57 | 1.35 |  | 1.65 | 2.26 |
| Four-horned antelope | 19.00 |  | 4 |  | 1.79 | 2.65 |  | 3.06 | 2.67 |  | 3.30 | 4.69 |
| Langur | 8.00 |  | 12 |  | 1.06 | 2.26 |  | 5.46 | 6.85 |  | 10.26 | 28.58 |
| Chevrotain | 5.00 |  | 2 |  | 0.74 | 2.16 |  | 0.63 | 1.09 |  | 1.83 | 7.27 |

^a^ data from [2]

**CF1 and CF2 of Indian wolf applied to dhole scats.** Application of CF1 and CF2 derived in this study from a feeding experiment with Indian wolves by [3] (see Table S1) to 165 dhole scats collected in the field by [2]. [2] used for their diet determination the conventional method with one CF based on the linear relationship *y* = 0.02*x*+0.38 derived from a wolf feeding experiment in North America [5] with *x* being the mean prey body mass provided per feeding experiment and *y* the consumed mean prey mass to excrete one collectable scat. Consumed prey mass to excrete one scat and the diet composition based on consumed mass and consumed number of prey individuals, respectively, were calculated with the new method and the conventional method. Dhole feeding group size was considered to be one and samples were considered to be collected dependently from each other.

| Prey |  |  | Scats with prey hair |  | Consumed mass to excrete 1 scat (kg) | |  | Diet composition based on consumed mass (%) | |  | Diet composition based on consumed individuals (%) | |
| --- | --- | --- | --- | --- | --- | --- | --- | --- | --- | --- | --- | --- |
| Species^a^ | Assumed weight (kg) ^a^ |  | *n*^a^ |  | New method | [2]^b^ |  | New  method | [2]^b^ |  | New  method | [2]^b^ |
| Gaur | 85.00 |  | 1 |  | 1.08 | 1.88 |  | 0.75 | 0.81 |  | 0.99 | 0.53 |
| Sambar | 62.00 |  | 16 |  | 1.05 | 1.78 |  | 11.62 | 12.25 |  | 14.34 | 8.56 |
| Chital | 48.00 |  | 121 |  | 0.93 | 1.48 |  | 77.97 | 77.05 |  | 77.44 | 68.48 |
| Wild pig | 37.00 |  | 12 |  | 0.65 | 1.00 |  | 5.37 | 5.16 |  | 3.98 | 8.14 |
| Muntjac | 20.00 |  | 4 |  | 0.46 | 0.78 |  | 1.28 | 1.34 |  | 0.92 | 3.28 |
| Four-horned antelope | 19.00 |  | 9 |  | 0.44 | 0.76 |  | 2.76 | 2.94 |  | 2.00 | 7.57 |
| Langur | 8.00 |  | 1 |  | 0.21 | 0.54 |  | 0.14 | 0.23 |  | 0.16 | 1.42 |
| Chevrotain | 5.00 |  | 1 |  | 0.13 | 0.48 |  | 0.09 | 0.21 |  | 0.16 | 2.02 |

^a^ data from [2]

^b^ data derived from [2]

**References**

1. Marker LL, Muntifering JR, Dickman AJ, Mills MGL, Macdonald DW (2003) Quantifying prey preferences of free-ranging Namibian cheetahs. S Afr J Wildl Res 33: 43-53.

2. Andheria AP, Karanth KU, Kumar NS (2007) Diet and prey profiles of three sympatric large carnivores in Bandipur Tiger Reserve, India. J Zool 273: 169-175.

3. Jethva BD, Jhala YV (2004) Computing biomass consumption from prey occurences in Indian wolf scats. Zoo Biol 23: 513-520.

4. Ackerman BB, Lindzey FG, Hemker TP (1984) Cougar food habits in Southern Utah. J Wildl Manage 48: 147-155.

5. Floyd TJ, Mech DL, Jordan PA (1978) Relating wolf scat content to prey consumed. J Wildl Manage 42: 528-532.
