# Supplementary material for: An Advanced Method to Assess the Diet of Free-Ranging Large Carnivores Based on Scats
Source: PLoS One. 2012 Jun 8;7(6):e38066. doi: 10.1371/journal.pone.0038066 (PMC3371055; doi:10.1371/journal.pone.0038066)
Supplement: Table S3 — Guidelines for feeding experiments to ensure the experiments simulate as closely as possible natural feeding situations. (DOC) [file pone.0038066.s004.doc]

**Table S3.** Guidelines for feeding experiments to ensure the experiments simulate as closely as possible natural feeding situations.

| Purpose of feeding experiment | Procedure |
| --- | --- |
|  |  |
| Number of carnivores feeding from a carcass reflects number of carnivores hunting and feeding in the wild | Small prey animals are given to single carnivores, large prey animals are given to a group of carnivores |
| Time the carnivores are allowed to feed reflects time the carnivores feed from a prey animal in the wild | Carcasses are removed according to the carnivore(s)’ feeding habits, e.g. shortly after finishing eating vs removal after several days^b^ |
| Carnivores receive sufficient intake from the prey species provided^a^. | Several carcasses are provided. Carcasses are given separately one by one to prevent carnivores from consuming only the best parts of each prey animal |

^a^ This is only a concern when small prey animals are provided

^b^ The former is applicable to carnivores that hardly ever come back to a kill after they have fed from it such as cheetahs [1], the latter to carnivores that might return to their kill for up to three days such as wolves [2] or lynx [3] .

**References**

1. Caro T (1994) Cheetahs of the Serengeti plains: group living in an asocial species. Chicago: University of Chicago Press. 478 p.

2. Jedrzejewski W, Jedrzejewska B, Okarma H, Schmidt K, Zub K, et al. (2000) Prey selection and predation by wolves in Bialowieza primeval forest, Poland. J Mammal 81: 197-212.

3. Nowicki P (1997) Food habit and diet of the lynx (Lynx lynx) in Europe. J Wildl Res 2: 161-166.
